# Dosing interval regimen shapes potency and breadth of antibody repertoire after vaccination of SARS-CoV-2 RBD protein subunit vaccine

**DOI:** 10.1038/s41421-023-00585-5

**Published:** 2023-07-28

**Authors:** Shuxin Guo, Yuxuan Zheng, Zhengrong Gao, Minrun Duan, Sheng Liu, Pan Du, XiaoYu Xu, Kun Xu, Xin Zhao, Yan Chai, Peiyi Wang, Qi Zhao, George F. Gao, Lianpan Dai

**Affiliations:** 1 Faculty of Health Sciences, University of Macau, Macau SAR, China; 2 Savaid Medical School, University of Chinese Academy of Sciences, Beijing, China; 3 CAS Key Laboratory of Pathogen Microbiology and Immunology, Institute of Microbiology, Chinese Academy of Sciences, Beijing, China; 4 Shenzhen Institute of Advanced Technology, Chinese Academy of Sciences, Shenzhen, Guangdong China; 5 Shenzhen Children’s Hospital, Shenzhen, Guangdong China; 6 School of Life Sciences, Yunnan University, Kunming, Yunnan China; 7 Department of Biology, Cryo-EM Center, Southern University of Science and Technology, Shenzhen, Guangdong China; 8 Vazyme Biotech, Nanjing, Jiangsu China; 9 Research Network of Immunity and Health (RNIH), Beijing Institutes of Life Science, Chinese Academy of Sciences, Beijing, China; 10 MoE Frontiers Science Center for Precision Oncology, Faculty of Health Sciences, University of Macau, Macau SAR, China

**Keywords:** Immunology, Structural biology

## Abstract

Vaccination with different vaccines has been implemented globally to counter the continuous COVID-19 pandemic. However, the vaccine-elicited antibodies have reduced efficiency against the highly mutated Omicron sub-variants. Previously, we developed a protein subunit COVID-19 vaccine called ZF2001, based on the dimeric receptor-binding domain (RBD). This vaccine has been administered using different dosing intervals in real-world setting. Some individuals received three doses of ZF2001, with a one-month interval between each dose, due to urgent clinical requirements. Others had an extended dosing interval of up to five months between the second and third dose, a standard vaccination regimen for the protein subunit vaccine against hepatitis B. In this study, we profile B cell responses in individuals who received three doses of ZF2001, and compared those with long or short dosing intervals. We observed that the long-interval group exhibited higher and broader serologic antibody responses. These responses were associated with the increased size and evolution of vaccine-elicited B-cell receptor repertoires, characterized by the elevation of expanded clonotypes and somatic hypermutations. Both groups of individuals generated substantial amounts of broadly neutralizing antibodies (bnAbs) against various SARS-CoV-2 variants, including Omicron sub-variants such as XBB. These bnAbs target four antigenic sites within the RBD. To determine the vulnerable site of SARS-CoV-2, we employed cryo-electron microscopy to identify the epitopes of highly potent bnAbs that targeted two major sites. Our findings provide immunological insights into the B cell responses elicited by RBD-based vaccine, and suggest that a vaccination regimen of prolonging time interval should be used in practice.

## Introduction

Severe acute respiratory syndrome coronavirus 2 (SARS-CoV-2), the virus responsible for causing coronavirus disease 2019 (COVID-19), continues to undergo evolutionary changes, resulting in the emergency of new variants of concern (VOCs). Among these, the Omicron variant (B.1.1.529) emerged rapidly since November 2021, leading to subsequent waves of infections worldwide^1^. Omicron variant is characterized by significant mutation in its spike (S) protein, which is a major target of vaccines. In particular, the receptor-binding domain (RBD) of the spike protein has undergone more than 15 amino acid substitutions, altering both the transmissibility and its recognition by the immune system^2,3^. Omicron variant contains several sub-variants that have emerged sequentially, including BA.1, to BA.2, BA.2.12.1, and currently circulating sub-variants BA.4/5, BF.7, BQ.1/1.1, and XBB. These sub-variants have shown reduced susceptibility to immunity induced by vaccines or previous infections^4–9^. To mitigate Omicron-related illnesses, vaccination strategies involving either primary immunization or booster doses with vaccines designed based on the ancestral virus or bivalent SARS-CoV-2 vaccines have been implemented globally^10^.

Globally, numerous COVID-19 vaccines employing different strategies have been administered, targeting three major viral components: the whole virus, full-length S protein, and RBD^11–13^. Infection with SARS-CoV-2 or immunization with whole virus-based inactivated vaccines or vaccines based on full-length spike protein, utilizing mRNA or adenovirus vector platforms, have demonstrated the ability to induce B cell responses against various SARS-CoV-2 variants, including Omicron sub-variants^14–20^. Previously, we developed a protein subunit COVID-19 vaccine called ZF2001, which utilized a tandem-repeat dimeric RBD as the immunogen^21,22^. In a phase 3 trial, ZF2001 exhibited an efficacy of 81.4% in short-term follow-up and 75.7% in long-term follow-up^23^. ZF2001 has received approval in countries such as China, Uzbekistan, Indonesia, Colombia, and Kenya, with more than 300 million doses administered worldwide. In China, a three-dose regimen of ZF2001 is recommended to be completed within a 6-month period following the initial priming dose. Some recipients received three doses of ZF2001 with a 1-month interval between each dose due to urgent clinical requirements, while others had an extended dosing interval between the second and third doses up to five months, following the standard regimen for protein subunit vaccines like hepatitis B vaccine. Serologic data suggested that individuals with a longer dosing interval exhibited enhanced antibody responses compared to those with a shorter dosing interval. Specifically, these individuals displayed increased potency and breadth of neutralizing antibodies against SARS-CoV-2 and its variants, including Omicron sub-variants^4,24,25^.

Based on the finding regarding serologic antibody responses, our study aimed to examine the landscape of B-cell receptor (BCR) induced by the RBD-based vaccine ZF2001 in individuals with different dosing intervals. Additionally, we aimed to investigate the breadth of vaccine-induced responses to major VOCs and Omicron sub-variants at the monoclonal antibody (mAb) level. The epitopes targeted by broadly neutralizing (bnAbs) were determined using competition assay and cryo-electron microscopy (cryo-EM). The data obtained from our study provide valuable immunological insights into the impact of dosing interval on the quality of B cell responses in the context of vaccination of an RBD-based COVID-19 vaccine. These findings suggest the need to optimize vaccination regimens in practice setting and highlight the important of developing future vaccines with a broader spectrum of protection.

## Results

### Serologic antibody responses

**Fig. 1:**
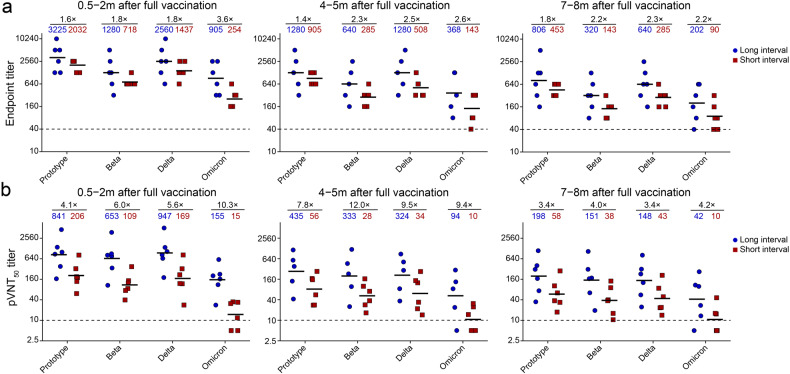
Serologic antibody responses. Serum samples were obtained from 12 donors who had received three doses of ZF2001 protein subunit vaccine with long or short dosing interval (*n* = 6 for each group) between dose 2 and 3. Detailed information of the participants and sample collection is described in Supplementary Fig. S1. The samples were tested for binding and neutralizing antibodies against SARS-CoV-2 prototype and VOCs (Beta, Delta, and Omicron BA.1). **a** Endpoint titers of serum IgG antibodies were tested by ELISA. **b** Serum neutralizing activity were tested as pVNT_50_. For (**a**) and (**b**), the values of geometric mean titers (GMTs) are shown by the short lines and the reduction folds are shown above scatters. The dashed lines indicate the lower limit of detection (LOD). The values lower than lower LOD were calculated as the half of LOD.

We analyzed the antibody responses in serum samples of 12 individuals after receipt of three doses of ZF2001 vaccine from a cohort recruited previously (Supplementary Fig. S1)^24^. Six individuals had received three doses of vaccine at month 0, 1, and 2 (short-interval group). The other six individuals had received three doses of vaccine at month 0, 1, and 4–6, with an extended time interval between the second and third dose (long-interval group). Serologic binding and neutralizing antibodies were measured at the following time points: 0.5–2 months, 4–5 months, and 7–8 months after the third dose. Consistent with our previous reports^4,24^, binding antibodies to RBD from prototype SARS-CoV-2 (ancestral virus identified in Wuhan) and its VOCs (Beta, Delta, and Omicron BA.1) were higher in the long-interval group in comparison to the short-interval group at all time points (Fig. 1a). Consistently, the titers of neutralizing antibodies against VSV-pseudotyped viruses which bear S proteins of prototype SARS-CoV-2 or its VOCs on the surface of virions were also higher in the long-interval group at all time points (Fig. 1b). Notably, the titer gap between these two groups widened when serum samples were tested for the binding and neutralization of pseudovirus from the cognate prototype SARS-CoV-2 to VOCs at all time points (Fig. 1a, b).

### B cell responses

**Fig. 2:**
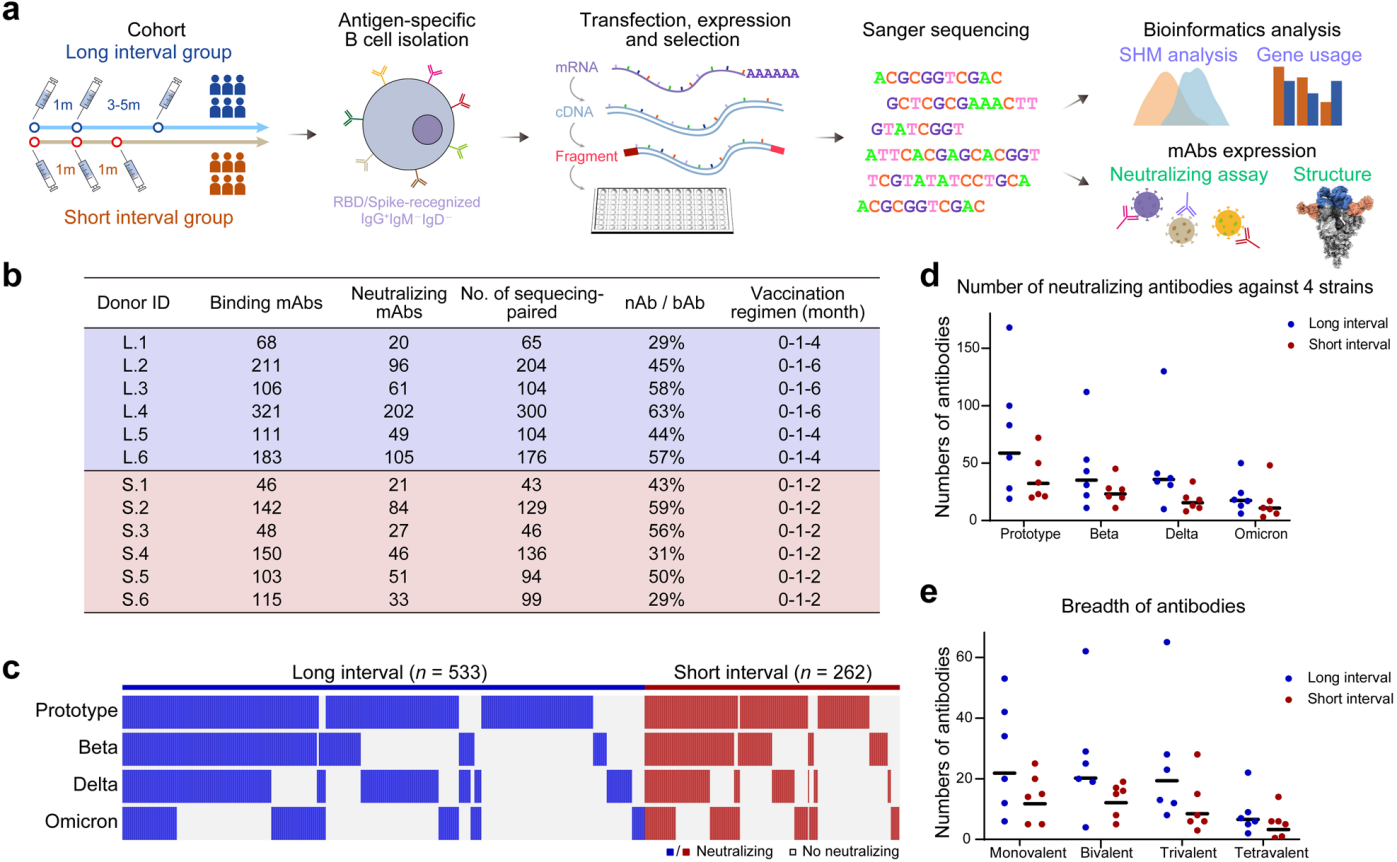
Study design for B cell profile and characteristic of mAbs. **a** Experimental design for analyzing the BCR repertoires. mAb, monoclonal antibodies. **b** Summary of antigen-binding mAbs recovered from B-cell clones of donors with long or short dosing interval of ZF2001 vaccine. “L” indicates that the volunteers belong to long-interval group and “S” indicates that the volunteers belong to short-interval group. mAb, monoclonal antibody; nAb, neutralizing antibody; bAb, antibody that can bind to prototype RBD. **c** Characterization of all mAbs with binding activity to prototype RBD. Each column represents a single mAb, with their neutralizing activity to prototype, Beta, Delta, and Omicron, respectively. mAbs with neutralizing activity are colored in blue (long-interval group) or in red (short-interval group). mAbs without neutralizing activity are colored in light gray in both groups. "*n*" on the top of graph represents the number of all binding mAbs in each group. **d** The number of neutralization mAbs against each strain. Data are the numbers of mAbs recovered from volunteers of long-interval group (blue) and short-interval group (red) that can neutralize SARS-CoV-2 prototype, Beta, Delta and Omicron BA.1. The lines show the GMTs of six volunteers. **e** The breadth of neutralizing mAbs. Monovalent, bivalent, trivalent and tetravalent in graph indicate that the antibody can neutralize one, two, three and all of four stains (prototype, Beta, Delta, Omicron BA.1), respectively. Data show the numbers of mAbs recovered from volunteers from long-interval group (blue) and short-interval group (red). The lines show the GMTs of six volunteers. One volunteer from short-interval group has no tetravalent neutralization mAb, with the antibody number calculated as 0.5.

To profile the BCR repertoires elicited by the RBD-based protein subunit vaccine ZF2001 with either long- or short-dosing interval, antigen-binding CD19^+^IgG^+^ B cells from both groups were sorted from PBMCs (Fig. 2a; Supplementary Fig. S2). The mRNA from each single B cell was extracted and reversed to cDNA. Variable regions of heavy (VH) and light (VL) chains were individually amplified and cloned into IgG1 backbone for antibody production. The supernatant of the cells that were transfected with DNA fragments of each mAb was screened for binding to prototype SARS-CoV-2 RBD. The positive supernatants were further tested for their neutralization breadth against pseudotyped HIV bearing S protein of prototype, Beta, Delta, and Omicron (BA.1), respectively.

As a result, 1604 mAbs obtained from B cell clones bound prototype RBD, with 1000 and 604 mAbs derived from the long- and short-interval individuals, respectively (Fig. 2b). Among them, 533 (long-interval group) and 262 (short-interval group) mAbs showed detectable neutralizing activity against prototype SARS-CoV-2 pseudovirus or its variants (Fig. 2b, c). Therefore, the long-dosing interval developed an increased number of B cells encoding antibodies with binding and neutralizing activities to SARS-CoV-2. In the long-interval group, 453 mAbs neutralized prototype pseudovirus, 272 neutralized Beta variant, 283 neutralized Delta variant, and 128 neutralized Omicron variant (BA.1). In contrast, in the short-interval group, 219 mAbs neutralized prototype pseudovirus, 152 neutralized Beta variant, 104 neutralized Delta variant, and 95 neutralized Omicron variant (BA.1). Substantial proportion (13.9%) of ZF2001-elicited RBD-binding antibodies neutralized Omicron variant. Compared with the short-interval individuals, the long-interval individuals were observed with higher geometric mean numbers of mAb in the neutralization of SARS-CoV-2 pseudoviruses, including its variants (Beta, Delta, and Omicron BA.1) (Fig. 2d, e). These results suggested the advantages of the extended dosing interval of ZF2001 vaccination in B cell quantity and quality.

**Fig. 3:**
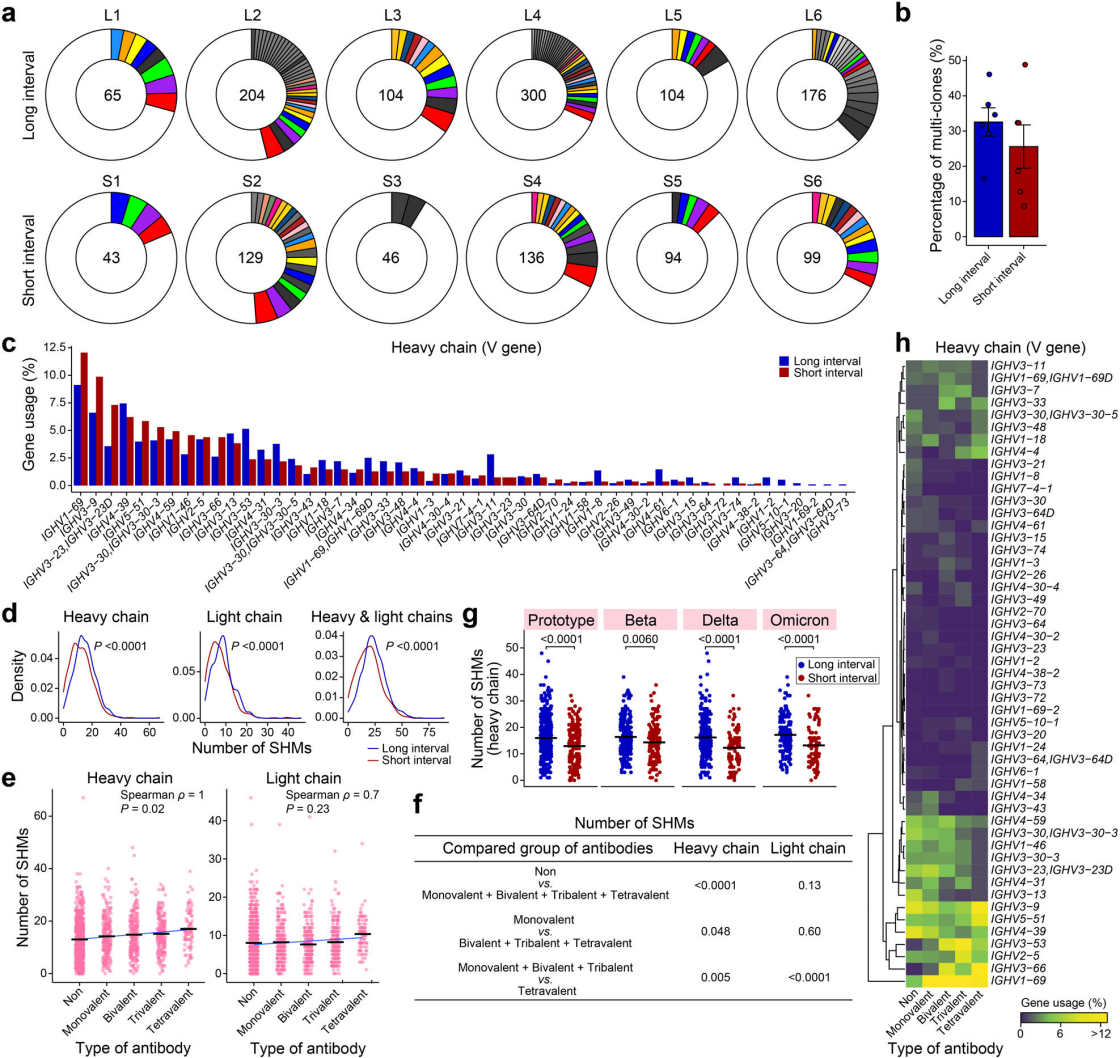
Analyses of BCR repertoires from vaccinees with different dosing intervals. **a** Pie charts showing distribution of RBD-binding mAb sequences obtained from B cells of the donors with long or short dosing interval of ZF2001 vaccine. The number inside each circle denotes the number of sequences analyzed for the donor indicated above. The pie slice size is proportional to the number of clonal relatives. The colored slices indicate expanded clonotypes using the same VH and VL genes. The gray slices indicate expanded clonotypes using different VH and VL genes. White slices indicate single clonotypes that occur only once in donor’s repertoire. **b** Percentage of expanded clones in donors with different dosing intervals, related to **a**. Data are the means ± SEM. **c** VH gene usage in RBD-binding mAbs obtained from B cells of the donors with long or short dosing interval of ZF2001 vaccine. **d** Somatic hypermutation (SHM) density plots of B cell-derived RBD-binding mAbs from the donors with the long or short dosing interval of ZF2001 vaccine. VH (left); VL (middle); VH and VL together (right). **e**, **f** SHM numbers of B cell-derived RBD-binding mAbs from donors with different neutralizing breadths (**e**) and statistical difference analysis (**f**). **g** SHM numbers of B cell-derived neutralizing mAbs from donors against four strains. **h** Heatmap and hierarchical clustering of VH gene usage frequencies in B cell-derived RBD-binding mAbs from donors with different neutralizing breadths. Data are the percentage of mAbs with the indicated VH gene per column.

Next, all antigen-binding mAbs obtained from B cell clones were Sanger-sequenced. As a result, we obtained 1500 paired antibody sequences from these 12 individuals (Fig. 2b). Expanded clonotypes of antigen-binding B cells were detected in all individuals (Fig. 3a). Notably, the long-interval individuals showed a higher percentage of expanded clonotypes compared to the short-interval individuals (32.6% vs 25.6%) (Fig. 3b). This result suggested that the prolonged dosing interval of vaccination was prone to increase the proportion of antigen-binding B cells in clonality from single clonotypes to expanded clonotypes, indicating a more advanced B-cell evolution.

The gene usage of the immunoglobulin variable region (V region) in these individuals vaccinated with RBD-based vaccine was similar to those vaccinated with full-length S-based vaccine (Fig. 3c; Supplementary Fig. S3a)^18^. *IGHV1-69*, *IGHV3-9*, and *IGHV4-39* were the most frequent VH genes used in the long-interval group, and *IGHV1-69*, *IGHV3-9*, and *IGHV3-23* were the most frequent VH used in the short-interval group (Fig. 3c). *IGKV1-39*, *IGLV3-21*, and *IGKV3-20* were the most frequent VL detected in both groups (Supplementary Fig. S3a).

The analysis of BCR sequences showed pronounced differences in somatic hypermutations (SHMs) between the long- and short-interval groups. The B cell-derived binding antibodies from the long-interval individuals had significantly (Wilcoxon-rank sum test *P* < 0.0001) elevated nucleotide mutations of VH and VL compared with those from the short-interval individuals, with a shift of the SHM number (Fig. 3d, e; Supplementary Fig. S3b). This result indicated that the prolonged dosing interval of vaccination promoted the maturation of antigen-binding B cells.

We next analyzed the SHM of BCR sequences of B cell-derived binding antibodies with different neutralization breadths to SARS-CoV-2. The nucleotide mutations in heavy chain of antibodies elevated sequentially from those with non-neutralizing activity (average 13.0) to those with monovalent (average 14.2), bivalent (average 14.8), trivalent (average 15.2), and tetravalent (average 17.0) activities (Fig. 3e). Significantly (*P* < 0.0001) higher number of SHMs in heavy chain was found in neutralizing antibodies vs non-neutralizing antibodies, in multivalent antibodies vs monovalent antibodies, and in tetravalent antibodies vs mono-to-tri-valent antibodies (Fig. 3e, f). In contrast, the nucleotide mutations in light chain were less pronounced between the B-cell-derived antibodies with different neutralization breadths, except the tetravalent antibodies (Fig. 3e, f). We also analyzed the complementary determining region 3 (CDR3) length of these antibodies and found a negative correlation between antibody breadth and CDR3 length of heavy chain (Supplementary Fig. S3e, f). Accordingly, the mean CDR3 length of heavy chain was lower (*P* = 0.04) in the long-interval group than that in the short-interval group (Supplementary Fig. S3c, d). Therefore, our data suggested the increased B-cell maturation, indicated by the higher number of SHMs and lower CDR3 length of heavy chain, was associated with the extended neutralization breadth to SARS-CoV-2. Accordingly, we found a significantly higher number of SHMs of both heavy and light chains for neutralizing antibodies in the long-interval group than that in short interval group against all four SARS-CoV-2 strains (Fig. 3g; Supplementary Fig. S3g). Also, a shorter CDR3 of heavy chain was found in the long-interval group (Supplementary Fig. S3h). These results explained the greater B cell breadth after prolonged dosing-interval of vaccination.

The analysis of VH gene usage of the antigen-specific B cells with different neutralization breadths to SARS-CoV-2 revealed that *IGHV3-33, IGHV4-4, IGHV1-24, IGHV1-58, IGHV3-53*, and *IGHV3-66* were more frequently used in the multivalent clones (Fig. 3h; Supplementary Fig. S3i).

### Vaccine-elicited bnAbs

**Fig. 4:**
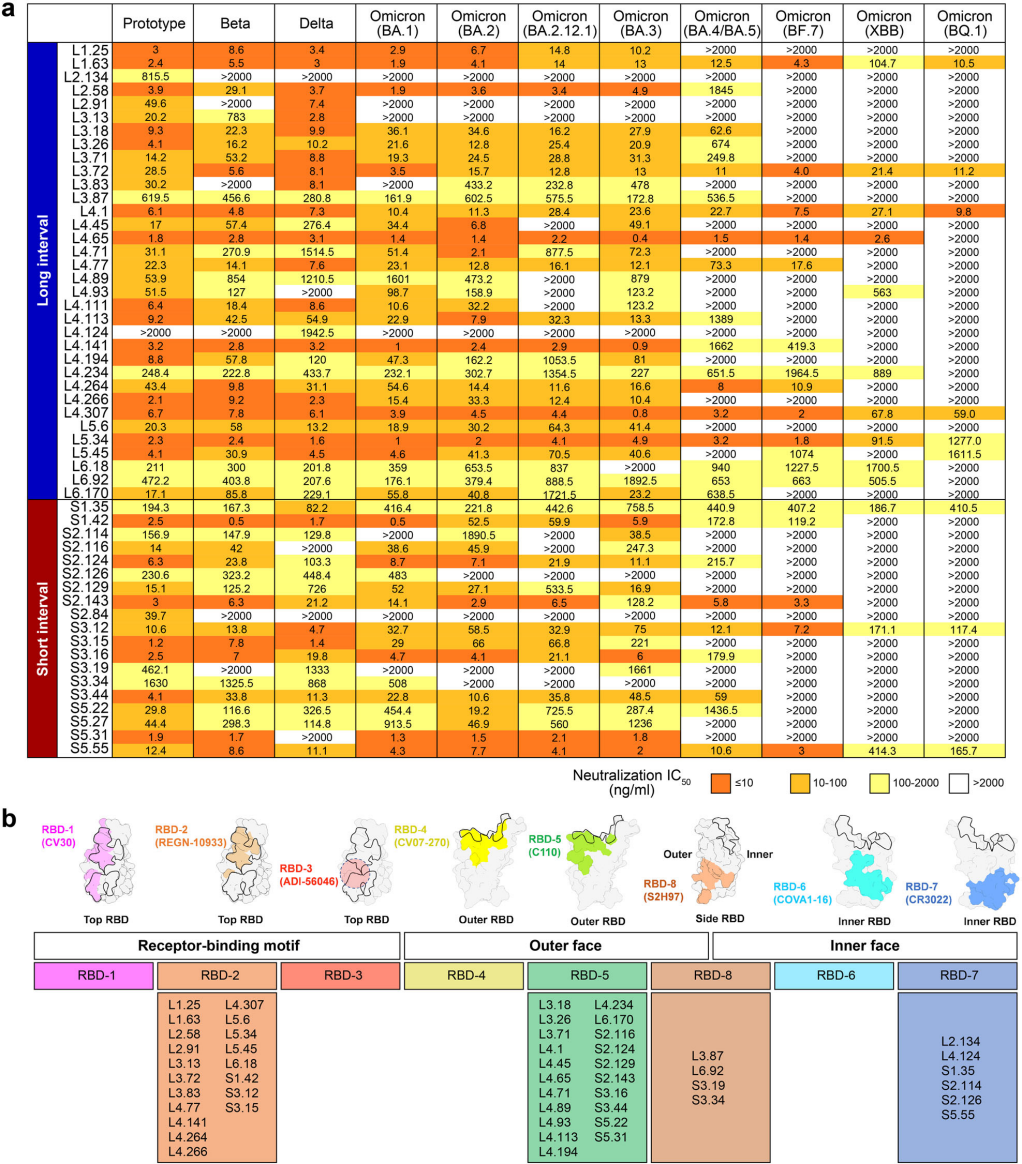
bnAbs and their epitope mapping. **a** 53 bnAbs (related to the tetravalent mAb in Fig. 2b) were expressed and purified as human IgG1. Antibody neutralization of pseudoviruses expressing S proteins of SARS-CoV-2 and its variants (prototype, Beta, Delta, and Omicron sub-variants, including BA.1, BA.2, BA.2.12.1, BA.3, BA.4/5, BF.7, XBB, and BQ.1) were tested as pVNT_50_. Shown is the heatmap according to neutralizing activity. Higher LOD was 2000 ng/mL. The antibodies were named as “Donor ID. B-cell number”. For example, mAb L1.25 means that the mAb was derived from the #25 B cell of Donor #L1. **b** Upper panel: footprints of representative benchmark antibodies from each antigenic site (RBD-1 to RBD-8) mapped onto a prototype SARS-CoV-2 RBD are highlighted in color. Structure models used for benchmark antibodies are as follows: CV30 (PDB: 6XE1); REGN-10933 (PDB: 6XDG); CV07-270 (PDB: 6XKP); C110 (PDB: 7K8V); COVA1-16 (PDB: 7JMW); CR3022 (PDB: 7JN5); S2H97 (PDB: 7M7W). The expected footprint of ADI-56046 was drawn as previously described^46^. RBM for hACE2 (PDB: 6LZG) is outlined with black lines. Bottom panel: antigenic sites of bnAbs grouped into clusters according to competition profile to eight benchmark antibodies (related to Supplementary Fig. S4).

We next cloned and expressed 65 tetravalent bnAbs as human IgG1, and successfully obtained 57 bnAbs. Among them, 53 bnAbs with fifty percent pseudovirus neutralization titer (pVNT_50_) value less than 50 μg/mL against prototype pseudovirus were further analyzed. The neutralizing activity for this panel of bnAbs was measured against pseudotyped VSV-expressing S protein from SARS-CoV-2 or its variants. Since a succession of Omicron sub-variants surged one another after the emerging of the BA.1, we tested the antibody neutralization to pseudotyped SARS-CoV-2 representing ancestral SARS-CoV-2 (prototype, Beta, and Delta) and Omicron sub-variants (BA.1, BA.2, BA.2.12.1, BA.3, BA.4/5, BF.7, XBB, and BQ.1). As a result, most of the bnAbs maintained the neutralizing potency to BA.2, and a few showed substantial reduction in neutralization of BA.2.12.1 and BA.3 (Fig. 4a). In contrast, most of the bnAbs largely reduced the neutralizing activity to BA.4/5, and only several of them still well neutralized (IC_50_ < 2000 ng/mL) the currently circulating BF.7, XBB, and BQ.1, suggesting the significant immune evasion. Notably, 10 of 34 (29.4%) bnAbs in the long-interval group neutralized XBB with IC_50_ < 2000 ng/mL, however, this ratio was decreased to 3/19 (15.8%) in the short-interval group. 6 of 34 (17.6%) bnAbs in the long-interval group neutralized BQ.1 with IC_50_ < 2000 ng/mL, with 3/19 (15.8%) in the short-interval group (Fig. 4a).

We next sought to determine the antigenic landscape of these bnAbs elicited by the RBD-based vaccine ZF2001. These bnAbs were first sorted by the competition profiles to nine benchmark mAbs that target seven major antigenic sites of SARS-CoV-2 RBD as defined previously (Fig. 4b; Supplementary Fig. S4)^26,27^. As a result, the epitopes of 19 bnAbs were mapped to the RBD-2 site, a place overlayed with receptor-binding motif (RBM) from the valley to the peak (similar to mAb REGN-10933); 21 were mapped to the RBD-5 site at the outer face beneath the mesa (similar to mAb C110 and S309); 6 were binned to the RBD-7 site at the inner face beneath the mesa (similar to mAb CR3022). In contrast, no epitope of bnAbs was mapped to RBD-1, -3, -4, and -6 sites. It is notable that four bnAbs (L3.87, L6.92, S3.19, and S3.34) were discovered not to compete with any of these nine benchmark mAbs, suggesting their different antigenic distributions. Nevertheless, they were all observed to compete with a pan-sarbecovirus mAb S2H97^28,29^, suggesting a new antigenic site (namely RBD-8 site) at the lateral ridge beneath the peak (Fig. 4b). Of note, the epitope binning only elucidates the bnAbs against four strains mentioned in our study, while the whole antibody repertoires may show different epitope maps.

### Structural analysis of ultrapotent bnAbs

Two bnAbs, L4.65 and L5.34, showed ultrapotent neutralizing activities (IC_50_ ≤ 10 ng/mL) against almost all the pseudoviruses tested (Fig. 4a). L4.65 and L5.34 were mapped to RBD-5 and RBD-2, respectively, the two major antigenic sites of bnAbs elicited by ZF2001 (Fig. 4b). To explore the molecular basis of their epitopes, we next determined the cryo-EM structures of these two bnAbs in complexed with S-trimer of prototype (resolution 2.65 Å), Omicron BA.2 (resolution 2.75 Å), and BA.4/5 (resolution 2.85 Å), respectively (Supplementary Table S1 and Figs. S5–S7).

**Fig. 5:**
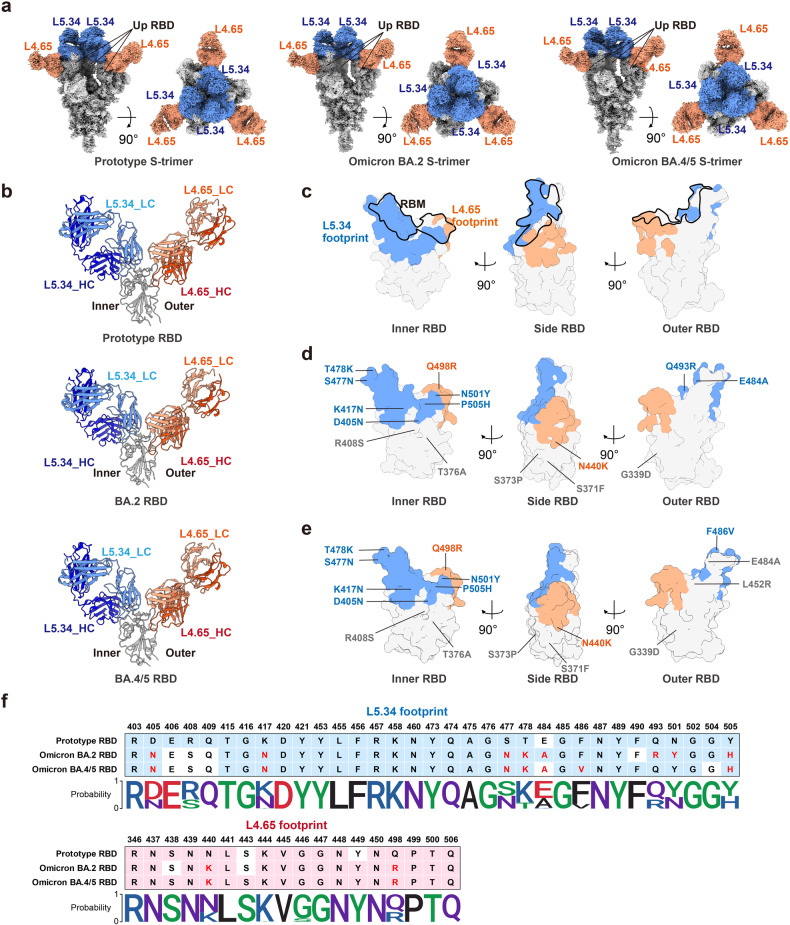
Structural basis of two representative ultrapotent bnAbs. **a** Side and top views of cryo-EM maps of SARS-CoV-2 S trimers (prototype, Omicron BA.2 and Omicron BA.4/5) in complexed with mAbs L5.34 and L4.65. S-trimer is colored in gray; L5.34 and L4.65 are colored in blue and orange, respectively. Three up RBDs are highlighted. **b** Cartoon representations of the structures of SARS-CoV-2 RBDs (prototype, Omicron BA.2 and Omicron BA.4/5) in complexed with mAb L5.34 and L4.65. The binding modes are shown as side view. Heavy chain is colored in dark blue (L5.34) or orange (L4.65), and light chain is colored in light blue (L5.34) or orange (L4.65). RBDs are colored in gray. **c**–**e** Footprint of mAb L5.34 (blue) and L4.65 (orange) on prototype RBD (**c**), BA.2 RBD (**d**), and BA.4/5 RBD (**e**). RBDs are shown as inner face, side face, and outer face. The black lines outlined the RBM. Amino acid variations in BA.2 and BA.4/5 are highlighted, with those in L5.34 footprint colored in blue, those in L4.65 footprint colored in orange, and those outside the footprints of these two mAbs colored in gray. **f** The interactive residues of L5.34 and L4.65 footprints on SARS-CoV-2 RBDs (prototype, Omicron BA.2 and Omicron BA.4/5). Cells colored in blue or pink represent the residues involved in the antibody interaction. Red characters highlight the mutated residues on the RBD of BA.2 or BA.4/5. Sequence conservation of the interactive residues of L5.34 and L4.65 footprints from 122,413 SARS-CoV-2 sequences randomly selected from the GISAID database representing the virus strains from Jan 2020 to Jan 2023. Sequence Logo plots illustrate the amino acid distributions at each interactive residue. Letter height represents frequencies of amino acid in dataset.

Notably, an S-trimer bound three pairs of fragments antibody-binding (Fabs) of L4.65 and L5.34 in its open state without steric clash (Fig. 5a). Three Fab pairs engaged three up RBDs with the same binding mode in different S-timers. L5.34 and L4.65 interact with the inner and outer face of the RBD, respectively (Fig. 5b). The footprint of L5.34 locates at the typical RBD-2 site towards RBD peak, largely overlapping with the RBM, while the footprint of L4.65 is at the typical RBD-5 site beneath the mesa in the outer face of RBD (Fig. 5c–e). The epitopes of L5.34 harbor more mutations in Omicron BA.2 (D405N, K417N, S477N, T478K, E484A, Q493R, N501Y, and Y505H) and BA.4/5 (D405N, K417N, S477N, T478K, E484A, F486V, N501Y, and Y505H), compared with the epitopes of L4.65 (N440K and 498R) in both Omicron sub-variants (Fig. 5c–f). This explained the largely decreased binding affinity of L5.34, but not L4.65, to RBD of Omicron BA.2 or BA.4/5, when compared with its binding to prototype RBD (Supplementary Fig. S8). Additionally, we analyzed SARS-CoV-2 sequences randomly selected from the Global Initiative on Sharing Avian Influenza Data (GISAID) database representing the virus strains up to Jan 2023, and found that the epitopes of these two bnAbs in prototype, Omicron BA.2, and BA.4/5 had covered most of amino acid variations occurred before (Fig. 5f), suggesting two vulnerable sites for broad neutralization of SARS-CoV-2.

## Discussion

This is a significant demand for rational vaccination administration to induce bnAbs against SARS-CoV-2 with different regimens. One potential strategy to meet this demand is by implementing a prolonged dosing interval. In phase 1, 2, and 3 clinical trials of ZF2001, the vaccine was administered using a three-dose vaccination regimen of three doses at month 0, 1, and 2, in response to the urgent clinical needs^23,30^. However, for protein subunit vaccines used to prevent diseases like hepatitis B and human papillomavirus infections, a more common practice is to have a prolonged dosing interval between the second and third immunizations, such as the 0–1–6 or 0–2–6 months regimen^31,32^. Notably, beneficial antibody responses have been observed in individuals receiving prolonged dosing intervals^4,24^. However, a booster immunization was required within 6 months of the initial priming after the approval of ZF2001 for use in China. This limitation prevents the collection of samples with even longer intervals between the second and third dose, and it is also challenging to recruit volunteers with a longer interval between the first and second dose. This study provided insights to explain this beneficial effect of prolonged dosing interval and support the use of the common vaccination regimen, such as the 0–1–6 months regimen used for HBV vaccination, for vaccination of COVID-19 protein subunit vaccines like ZF2001. Similarly, a previous study showed an extended dosing interval (6–14 weeks) of mRNA vaccines induced higher levels of neutralizing antibodies and IL2^+^ CD4^+^ T cells compared with the standard intervals (3–4 weeks)^33^.

An extended dosing interval is expected to provide a longer duration for B-cell maturation before administering the booster vaccination, leading to an enhanced antibody response following the boosting. Additionally, it has been observed that high titer of circulating antibodies from primary responses can inhibit the recruitment of cognate naïve B cells to germinal centers (GCs) during secondary responses^34^. Consequently, a recent study has shown that pre-existing antibodies can altered B cell responses to SARS-CoV-2 vaccination in human^35^. By extending the interval between the second and third dose of ZF2001, there is a longer period of waning in circulating antigen-specific antibodies. This extended interval allows for enhanced recruitment of cognate naïve B cells to GCs after the third dose, promoting the evolution of the antigen-specific B cells and potentially improving the quality of the immune response.

Previous studies have provided evidence that antigen-experienced B cells with higher affinity, and tend to have shorter CDR3 of the heavy chain^36,37^. This is because antibodies with long CDR3s have limited space in the antibody-binding pocket, making it more challenging for antigens to fit^36^. Thus, it is likely that both VDJ joining (which can lead to CDR3 shortening) and SHMs during the antibody maturation contribute to the development of antibodies with higher affinity. These affinity-enhanced antibodies are more likely to recognize the common epitopes shared by spike proteins of different variants. In our study, we found that the CDR3 length of light chain is not associated with the breadth of neutralization (Supplementary Fig. S3e, h). It is believed that the light chain has a lesser contribution to the antigen-binding affinity compared to the heavy chain^38^. Additionally, observations have shown that the length of CDR3 in light chain does not vary significantly between mutated and non-mutated antibodies^39^. This may be due to the fact that the optimal length of the light chain CDR3 to bind to antigens is typically around 6–12 amino acid^36^.

The study identified RBD-2 and RBD-5 as the two major sites for ZF2001-elicited bnAbs. Notably, RBD-2-directed bnAbs were predominately found in individuals with a prolonged interval (Fig. 4b), whereas bnAbs targeting the other sites (RBD-5, -7, and -8) were more balanced distribution between the long- and short-interval groups. Previous observations have indicated that most neutralizing antibodies targeting RBD-2 lose binding and neutralization capabilities against Omicron sub-variants due to the numerous mutations in RBM^27^. However, in this study, it was found that extending the dosing interval promoted the maturation of antigen-binding B cells, which is believed to enhance the antibody affinity. This increased affinity may help mitigate the reduction of neutralization to Omicron sub-variants by buffering the impact of viral mutations on for RBD-2-directed bnAbs. As shown in Supplementary Fig. S8, we demonstrate that the binding affinity of RBD-2-directed mAb L5.34 to RBD of Omicron BA.2 or BA.4/5 strains has a huge reduction (1–2 orders of magnitude) compared with that to prototype RBD. However, despite this reduction, the binding affinity (*K*_D_ of 1.67E^−10^ M and 4.07E^−10^ M to bind BA.2 and BA.4/5, respectively) remains relatively high. The high affinity is still effective in efficiently preventing the viral infection. These findings support the hypothesis that antibody maturation enhances the overall binding affinity to the prototype RBD, which helps buffer the decrease of binding and neutralization caused by the viral mutation (Fig. 4b; Supplementary Fig. S8).

In summary, our study characterized the B cell responses in individuals who received the RBD-based COVID-19 vaccine and yielded important immunological insights into the enhanced potency and breadth of antibodies achieved with a prolonged dosing interval. The landscape of vaccine-induced bnAbs identified in this study can provide guidance for the development of new vaccines with broader coverage. Notably, the identification of ultrapotent bnAbs, including L4.65 and L5.34, highlight their potential as therapeutic candidates for the treatment of SARS-CoV-2 and its variants.

## Materials and methods

### Participants and samples

Blood samples were obtained from 12 volunteers after receipt of 3 doses of ZF2001 vaccine: 6 of them received vaccine at month 0, 1, and 2; 3 of them received vaccine at month 0, 1, and 4; the other three received vaccine at month 0, 1, and 6. These blood samples were collected at three time points: 1) 0.5–2 months after the third dose, 2) 4–5 months after the third dose, 3) 7–8 months after the third dose. All participants provided written informed consent for the collection of information, storage, and usage of their samples for research purpose, and publication of data generated from this study. Detailed participant characteristics were shown in Supplementary Fig. S1.

Whole blood samples in separate gel procoagulant tubes were centrifuged to isolate sera. Peripheral Blood Mononuclear Cells (PBMCs) were isolated from whole blood by Lympohoprep^TM^ (STEMCELL, 07851) and SepMate^TM^ -50 (STEMCELL, 86450). Briefly, PBMCs were collected from the upper layer after centrifugation, and washed by PBS with 2% fetal bovine serum (FBS) (Gibco) twice. Cryopreserved PBMCs were thawed in CryoStor CS10 (STEMCELL, 07930) before usage.

### Ethics statement

This study was approved by the Ethics Committee of the Institute of Microbiology, Chinese Academy of Sciences (SQIMCAS2021149). All candidates signed the written informed consent.

### Cells

HEK293-ACE2 cells (Vazyme, DD1401) were maintained in OPM-293 CD05 Medium (OPM, 81075-001) at 37 °C under 5% CO_2_. Vero-E6 cells (ATCC, CRL-1586) were maintained in Dulbecco’s modified Eagle’s medium (DMEM, Invitrogen, USA) supplemented with 10% FBS at 37 °C under 5% CO_2_. Expi293F^TM^ cells (Thermo Fisher Scientific) were cultured in SMM 293T-II (Sino Biological, M293TII) at 37 °C under 5% CO_2_.

### Isolation of antigen-specific B cell

EasySep^TM^ Human B Cell Isolation Kit (Stemcell, 17954) was used to negatively select B cells. Then 1 × 10^6^ B cells were stained with 5 μL PE conjugated anti-human IgG Fc antibody (BioLegend, 410708), 5 μL PerCP/Cyanine5.5 conjugated anti-human IgM antibody (BioLeged, 314512), 5 μL PerCP/Cyanine5.5 conjugated anti-human IgD antibody (BioLeged, 307810) and AF488 conjugated antigens, which consist of a mixture of RBD monomer, RBD dimer and spike proteins of SARS-CoV-2 (prototype, HB-01 strain) that were labeled by Alexa Fluor® 488. Target cells (IgM^–^IgD^–^IgG^+^ antigen^+^) were sorted by cell sorter (SONY, sh800s) to V-bottom plates containing lysis buffer.

### Variable (V) region amplification and mAb production

This process is similar with the Mouse Single Cell BCR IgG H/K Amplification Kit (DD5101). In brief, the first chain of DNA was synthesized using mRNA as the template with a 5’ RACE label in the end. With the help of 5’ RACE label, cDNA was amplified and then the variable (V) region of antibodies was amplified by nested PCR. The V region of both heavy and light chain were then linked respectively with human IgG1 skeleton as DNA fragment for monoclonal antibody (mAb) production.

1.6 μg DNA fragments were mixed with FectoPRO (Polyplus) and added into 1 × 10^6^ Expi293F^TM^ cells per mL. Three days after transfection, cells were centrifuged and the supernatants containing the secreted mAbs were collected.

### Enzyme-linked immunosorbent assay (ELISA)

Binding properties of antibodies to SARS-CoV-2 RBD protein were determined by ELISA as previously described, with some modifications. 96-well plates (CORNING, 3590) were coated overnight with 3 μg/mL of SARS-CoV-2 prototype, Beta, Delta or Omicron (BA.1) RBD protein in 0.05 M carbonate-bicarbonate buffer, pH 9.6, and blocked in 5% skim milk in PBS. mAb or supernatant containing the secreted mAb was diluted and added to each well. The plates were incubated for 1 h at room temperature and then washed. The plates were incubated with goat anti-human-IgG-HRP antibody, incubated for 1 h and then washed. The plates were subsequently developed with 3,3’,5,5’-tetramethylbenzidine (TMB) substrate. Reactions were stopped with 2 M hydrochloric acid, and the absorbance was measured at 450 nm using a microplate reader (PerkinElmer, USA). The positive sample was defined as the value beyond 1.

### V region sequencing

Single-direction Sanger sequencing was applied to the PCR products that amplified from cDNA of those mAbs with positive binding to prototype RBD. All the primers used for sequencing were inhouse from Vazyme.

### Pesudovirus neutralization

Two pesudovirus systems were used in this study: 1) HIV backbone expressing luciferase and 2) VSV backbone expressing GFP. The HIV-backbone pseudovirus encodes spike gene from prototype SARS-CoV-2 (Vazyme, DD1402), Beta variant (Vazyme, DD1441), Delta variant (Vazyme, DD1754) or Omicron variant (BA.1) (Vazyme, DD1768), and was used to screen neutralization positive mAbs secreted from Expi293F^TM^ cells transfected with DNA fragment coding for each mAb in Vazyme company. In brief, pesudovirus was diluted in DMEM to 1–2 × 10^4^ TCID_50_/mL. 90 μL pesudovirus was mixed with 90 μL 5-fold diluted supernatant from transfected cells and incubated for 1 h at 37 °C. Virus controls were 90 μL DMEM mixed with 90 μL pesudovirus. Blank controls were 180 μL DMEM. Negative control only contained transfection reagent without DNA fragment of mAb. HEK293-ACE2 cells were cultured overnight to 2 × 10^6^ cells/mL and 50 μL cells were cultured with 50 μL pseudovirus/supernatant mixture. 20–24 h later, 25 μL DMEM containing 10% FBS was added. After 48 h incubation, 100 μL Bio-Lite Luciferase Assay System (Vazyme, DD1201) was added and the RLU value was read. Inhibition rate = [1 – (RLU_sample_ – RLU_blank_)/(RLU_virus_ – RLU_conrol_ – RLU_blank_)] × 100%. Neutralization positive mAb was defined as the inhibition rate ≥ 60% and more than 30% of higher than that of the negative control. The VSV-GFP pesudovirus was produced as described before^22^ and used to test neutralizing activity for the vaccine-elicited sera and mAb in Institute of Microbiology. The methods for preparing pseudoviruses and neutralization assays were described previously. Briefly, purified antibodies were twofold serially diluted from 4 μg/mL and incubated with pseudovirus at 37 °C for 1 h. Then the mixture was transferred to pre-plated Vero-E6 cell monolayers in 96-well plates. After 16 h of incubation, the transducing unit numbers were calculated on a CQ1 confocal image cytometer (Yokogawa). pVNT_50_ was determined by fitting nonlinear regression curves using GraphPad Prism and calculating the reciprocal of the serum dilution required for 50% neutralization of infection. pVNT_50_ below the limit of detection shows > 2000 in Fig. 4a.

### Antibody sequences processing

Raw antibody sequences in FASTA format were aligned into human VDJC reference (IMGT domain delineation system) using software *igblastn* (version: 1.15.0). Then, antibody clonotypes were defined and annotated based on CDR3 sequence using function *MakeDb.py* and *DefineClones.py* in toolkit *Change-O* (version: 0.4.5).

Only antibodies with similar CDR3 sequence (Hamming distance greater than 85%) were considered as the same clonotype. If one clonotype occurred only once, this clonotype was defined as the single clone, and otherwise, as the expanded clonotype; in other words, each single clone corresponded to only one antibody. For a given expanded clonotype, if all antibodies had the same IGHV and IGLV genes, this clonotype was defined as the expanded clonotype with the same gene pair (corresponding to the colored pie in Fig. 3a), and otherwise, as the expanded clonotype with different gene pairs (corresponding to the gray pie in Fig. 3a).

### Calculating somatic hypermutations

The file *H/L_db-pass_clone-pass.tab* was used to calculate SHMs. We calculated nucleotide SHMs for each antibody based on the columns *SEQUENCE_IMGT* and *GERMLINE_IMGT* using function *observedMutations* in R package *shazam* (version: 1.1.0). And the statistical significance was obtained based on Wilcoxon-rank sum test.

### Protein expression and purification

All the constructs were transiently transfected into Expi293F^TM^ cells by Sinofection Transfection Reagent (Sino Biological, STF02) and cultured for 5 days.

The V regions of human mAbs were constructed on pCAGGS plasmid containing coding sequences for human IgG1 heavy chain or light chain. Antibodies were purified by AmMag^TM^ Protein A MagBeads (Genscript, L00672). Briefly, antibodies were combined with the beads, washed by AmMag^TM^ Wash buffer (Genscript, B00045) and eluted by 0.1 M Glycine. Medium was changed to PBS through centrifuge in concentration tubes (Merck millipore, UFC805008).

The coding sequences of SARS-CoV-2 prototype spike (GISAID: EPI_ISL_402119), Omicron BA.2 spike (GISAID: EPI_ISL_9652748), and Omicron BA.4/5 with a C-terminal 6× His tag and six mutations (F817P, A892P, A899P, A942P, K986P, V987P) were cloned into the pCAGGS vector, respectively. The cell supernatants expressing spike proteins were collected and purified by Ni affinity chromatography using a HisTrap^TM^ HP 5 mL column (Cytiva). The samples were further purified via gel filtration chromatography with Superose 6 Increase (Cytiva).

### Antibody affinity

Biolayer interferometry assays were performed on Octet RED 96 Protein Analysis System (Fortebio) according to the manufacturer’s instructions. To measure the binding affinities, mAbs were immobilized onto AHC biosensors (Fortebio) and the 2-fold serial dilutions of prototype RBD, BA.2 RBD and BA.4/5 RBD in PBST (0.05% v/v Tween 20 added into PBS) were used as analytes. Data were collected with Octet Acquisition 9.0 (Fortebio) and analyzed by Octet Analysis 9.0 (Fortebio).

### Antibody competition assay

Antibody competition assay was performed on Octet RED 96 Protein Analysis System (Fortebio) according to the manufacturer’s instructions. To measure the competition of two antibodies, biotinylated prototype RBD was immobilized onto SA biosensors (Fortebio). Biosensors combined the target antibodies and a site-known antibody in sequence. Data were collected with Octet Acquisition 9.0 (Fortebio) and analyzed by Octet Analysis 9.0 (Fortebio).

### Cryo-EM sample preparation and data acquisition

For the Prototype S-L4.65-L5.34, BA2 S-L4.65-L5.34, and BA4 S-L4.65-L5.34 complexes, C-flat R 2/1 holey carbon grids were first glow discharged for 20 s using a Pelco easiGlow glow discharge unit (Pelco easiGlow) and then 3.5 μL sample was applied to the surface of the grid at temperature of 5 °C and humidity level of 95%. Grids were then blotted for 1.5 s before being plunge-frozen in liquid ethane using Vitrobot Mark IV. Grids were imaged under 300 kV Titan Krios electron microscope (Thermo Fisher Scientific; one of microscope is equipped with selectris energy filter and Falcon4 direct electron detector (Thermo Fisher Scientific), and another microscope is equippted with Bio-Quantum energy filter and K3 direct electron detector (Gatan). Automatic data collection was performed using EPU software. Images were recorded at pixel size of 0.84 Å, 0.84 Å, or 0.856 Å for the dataset of BA2 S-L4.65-L5.34, BA4 S-L4.65-L5.34, and Prototype S-L4.65-L5.34, respectively. The exposure was performed with a dose rate of 15 e^-^/pixel/s and an accumulative dose of ~50 e^−^/Å^2^ for each image stack which was fractionated into 32 frames. The final defocus ranges of the datasets were ~–(1.0–2.0) μm.

### Image processing and 3D reconstruction

For the Prototype S-L4.65-L5.34 sample, the dose-fractionated image stacks were subjected to beam-induced motion correction using MotionCor2^40^. Initial contrast transfer function (CTF) values for each micrograph were calculated with CTFFIND4^41^. Micrographs with an estimated resolution limit lower than 4 Å were discarded in the initial screening. A set of ~150,000 particles were blob-picked and subjected to 2D classification to generate templates for auto-picking against the entire dataset. The subsequent image processing and reconstruction were performed using cryoSPARC^42^. 3,876,449 particles were picked from 13,805 micrographs. Then the picked particles were extracted and subjected to two rounds of reference-free 2D classification in cryoSPARC, which yielded 1,339,302 particle projections. This subset was subjected to one round of hetero refinement and the complex subsets were used to obtain a map with a resolution of 2.8 Å. This portion of particle projections was imported to cryoSPARC for further 3D classification without alignment. The predominant class containing a subset of 615,800 best particles shows the clear features of secondary structural elements. These particles were subjected to a non-uniform refinement, which yielded a reconstruction at 2.65 Å resolution. Local refinement focused on the RBD-L4.65-L5.34 with mask could reconstitute the structure at a 2.70 Å resolution (Supplementary Fig. S5). Local resolution estimate was performed with cryoSPARC.

The BA2 S-L4.65-L5.34 and BA4 S-L4.65-L5.34 data were processed similarly to the abovementioned Prototype S-L4.65-L5.34 data. The detailed image processing and reconstruction were shown in Supplementary Figs. S6 and S7, respectively.

### Model building

The structure of the spike protein (PDB: 6XCN), was docked into the cryo-EM density maps of the Prototype S-L4.65-L5.34, BA2 S-L4.65-L5.34, and BA4 S-L4.65-L5.34 complexes using CHIMERA^43^. The models were manually corrected for local fit in COOT^44^ and the sequence register was updated based on alignment. The models were refined against corresponding maps in real space using PHENIX^45^, in which the secondary structural restraints and Ramachandran restrains were applied. The stereochemical quality of each model was assessed using MolProbity. Statistics for model refinement and validation are shown in Supplementary Table S1.

## Supplementary information

Supplementary Information

## Data Availability

Coordinates and structure factors of the cryo-EM structures of Fabs of L4.65 and L5.34 in complex with spike proteins or RBDs reported here have been deposited into the Protein Data Bank (PDB) under accession codes: 8HQ7 (EMD-34946) (Prototype RBD); 8HPV (EMD-34945) (Prototype S-trimer); 8HPF (EMD-34931) (Omicron BA.2 RBD); 8HP9 (EMD-34928) (Omicron BA.2 S-trimer); 8HPU (EMD-34944) (Omicron BA.4/5 RBD); 8HPQ (EMD-34940) (Omicron BA.4/5 S-trimer), respectively. All the other data supporting the finding of this study are available within the paper and are available from the corresponding author upon request.
