# Supplementary material for: Dosing interval regimen shapes potency and breadth of antibody repertoire after vaccination of SARS-CoV-2 RBD protein subunit vaccine
Source: Cell Discov. 2023 Jul 28;9:79. doi: 10.1038/s41421-023-00585-5 (PMC10382582; doi:10.1038/s41421-023-00585-5)
Supplement: Supplementary file 1 — Supplementary Information [file 41421_2023_585_MOESM1_ESM.pdf]

Supplementary Materials for

Dosing interval regimen shapes potency and breadth of antibody repertoire after vaccination of SARS-CoV-2 RBD protein subunit vaccine

Shuxin Guo[1,2,#], Yuxuan Zheng[3,#], Zhengrong Gao[4,5,#], Minrun Duan[6,#], , Sheng Liu[7,#], Pan Du[8], XiaoYu Xu[8], Kun Xu[9], Xin Zhao[3], Yan Chai[3], Peiyi Wang[7], Qi Zhao[10], George F. Gao[2,3,9]*, Lianpan Dai[3,11]*

#These authors contributed equally to this work

*Corresponding author. Email: gaof@im.ac.cn (G.F.G.); dailp@im.ac.cn (L.D.)

This file includes:

Supplementary Figs.S1-S8

Supplementary Table S1

Supplementary Fig. S1

a

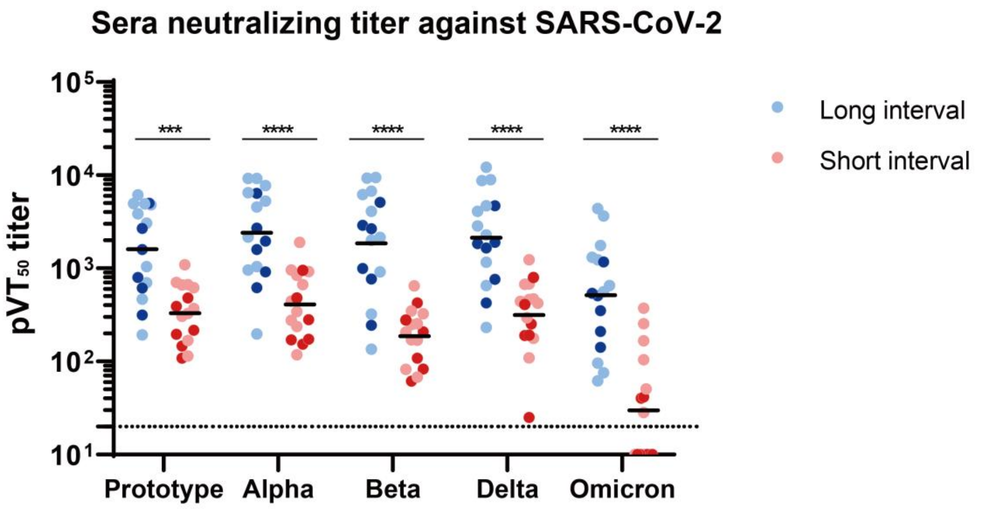

b

Groups	Long interval	Short interval
**No. of participants**	6	6
**Age (median, range)**	31 (26-38)	30 (24-37)
**Sex**		
Male (%)	2 (33.3)	2 (33.3)
Female (%)	4 (66.6)	4 (66.6)
**Time interval between the first and second doses (Days, median, range)**	33 (28-33)	28 (28-29)
**Time interval between the second and third doses (Days, median, range)**	135 (81-135)	35 (35-35)
**Time interval 1 between the third dose and blood sampling (Days, median, range)**	30 (27-57)	14 (14-14)
**Time interval 2 between the third dose and blood sampling (Days, median, range)**	125 (125-166)	122 (122-122)
**Time interval 3 between the third dose and blood sampling (Days, median, range)**	242 (218-259)	224 (224-224)

Supplementary Fig. S1: Blood donors and sampling

a The 32 volunteers from the long- (blue) and short-interval group (red) (n = 16 for each group) are shown in dots. The 12 volunteers (n = 6 for each group) recruited in this study are shown in dark. Two tailed Mann-Whitney test P values are indicated (***P < 0.001; ****P < 0.0001). **b** Demographic characteristics of the blood donors and the timetable of vaccination and blood sampling. L1-L6 are donors with long dosing interval of vaccination, while S1-S6 are donors with short dosing interval of vaccination.

Supplementary Fig. S2

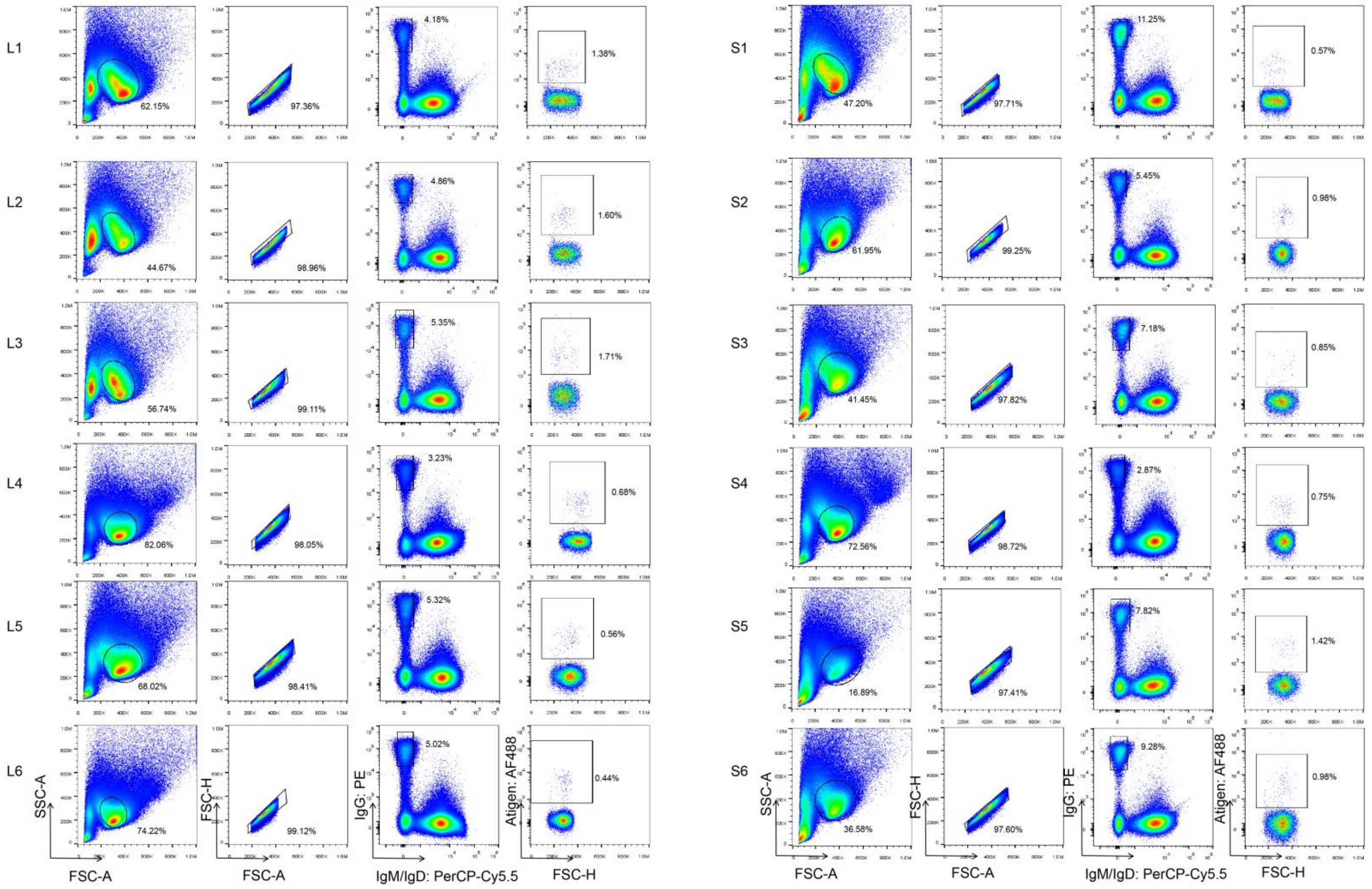

Supplementary Fig. S2: FACS strategy to isolate antigen-specific B cells from SARS-CoV-2-vaccinated PBMCs, related to Figure 1 and 2

Figure shows the sorting flow of each volunteers' B cell. The IDs of volunteer show behind the graphs. The left part shows the long interval group volunteers' cell sorting flow while the right part shows the short interval group volunteers'. Each picture shwos a 96-plate sorting result.

Supplementary Fig. S3

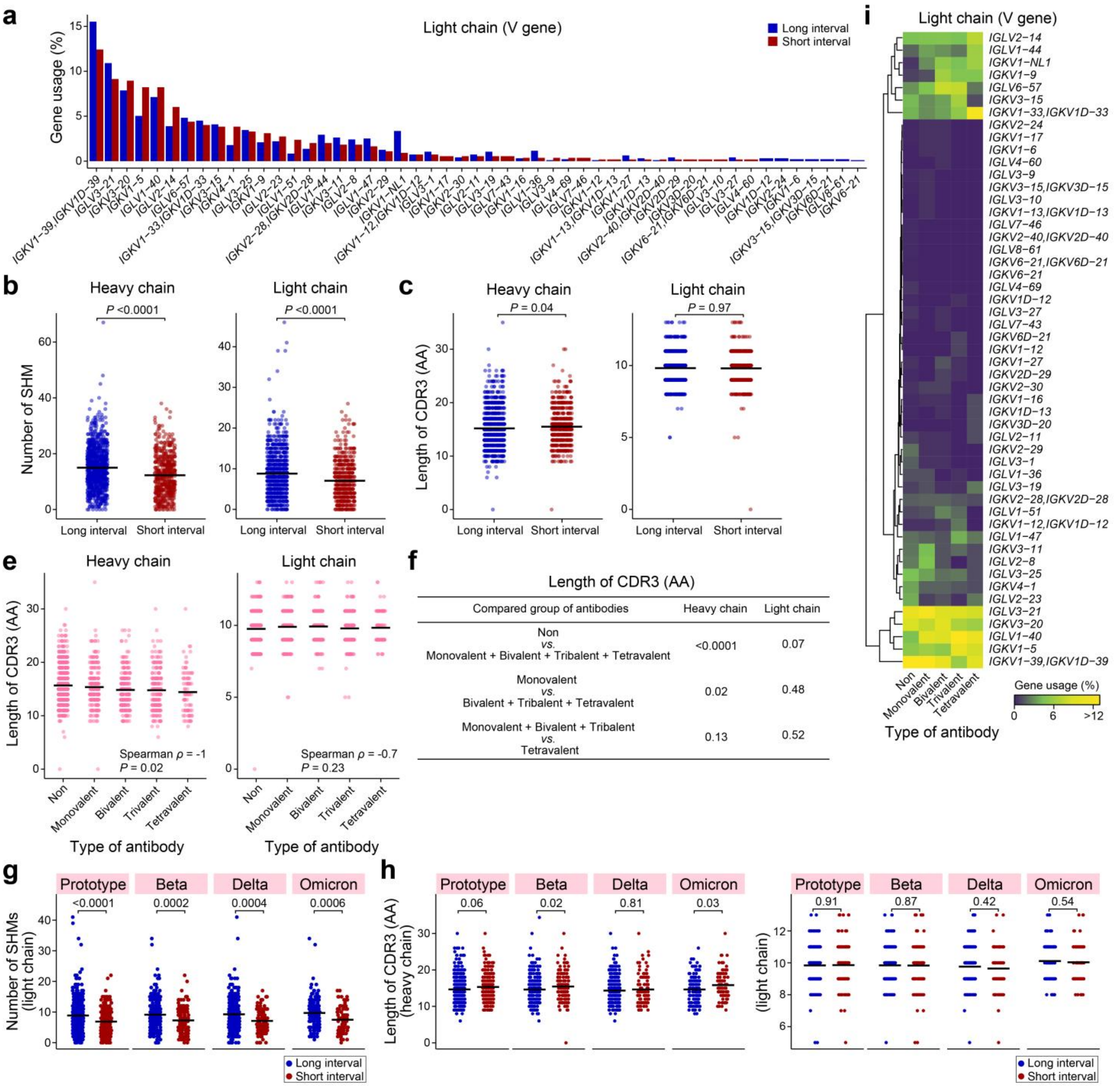

Compared group of antibodies	Heavy chain	Light chain
Non *vs.* Monovalent + Bivalent + Tribalent + Tetravalent	<0.0001	0.07
Monovalent *vs.* Bivalent + Tribalent + Tetravalent	0.02	0.48
Monovalent + Bivalent + Tribalent *vs.* Tetravalent	0.13	0.52

Supplementary Fig. S3: Analyses of B-cell repertoires from vaccinees with different dosing intervals

a Bar graph showing the VL gene usage in RBD-binding mAbs from B-cell clones of the donors in two groups. **b** Scatter plots showing the number of somatic hypermutation (SHM) of RBD-binding mAbs in heavy (left) and right (right) chains in two groups. Wilcoxon-rank sum test P values are indicated. **c-d** Scatter plot showing the length of CDR3 (amino acids) in heavy (**c**) and light chains (**d**) in two groups. Wilcoxon-rank sum test P values are indicated. **e-f** Scatter plot showing the length of CDR3 (amino acids) in heavy (left) and light (right) chains along with different neutralizing breadths (**e**). The corresponding statistical analysis results are indicated (**f**). **g** Scatter plots showing the length of CDR3 of neutralizing antibodies against four

strains in light (g) chains from the long- (blue) and short-interval groups (red). Wilcoxon-rank sum test P values are indicated. **h** Scatter plots showing the length of CDR3 (amino acids) of neutralizing antibodies against four strains in heavy (left) and light chains (right) from long- (blue) and short-interval groups (red). Wilcoxon-rank sum test P values are indicated. **i** Heatmap showing VL gene usage in RBD-binding mAbs along with different neutralizing breadths.

Supplementary Fig. S4

a

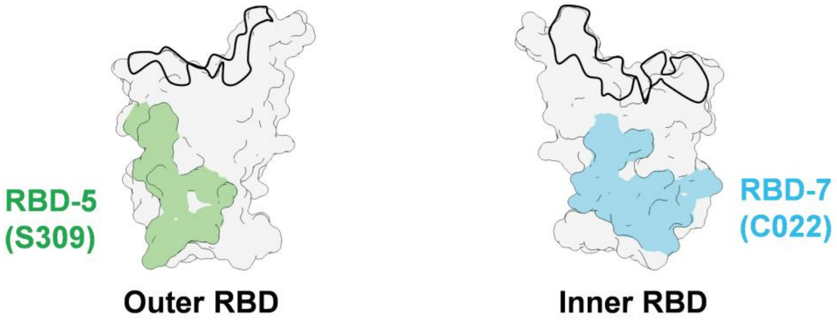

b

	RBD-1	RBD-2	RBD-3	RBD-4	RBD-5		RBD-6		RBD-7	RBD-8
	CV30	10933	ADI-56046	CV07-270	C110	S309	COVA1-16	C022	CR3022	S2H97
L1.25	0.0541	0.0755	0.9095	0.3746	0.333	0.4698	0.0952	0.271	0.8584	/
L1.63	0.0545	0.0834	0.9405	0.3725	0.3049	0.972	0.0993	0.362	0.9104	/
L2.134	0.702	0.7535	0.7738	0.572	0.7524	0.855	0.3005	0.398	0.3591	0.5543
L2.58	0.042	0.0582	0.8836	0.3368	0.396	0.8268	0.0843	0.267	0.9173	/
L2.91	0.1677	0.2627	0.2626	0.5401	0.878	0.8841	0.1717	0.423	0.8788	/
L3.13	-0.0271	0.0634	0.3099	0.8393	0.8263	0.7352	0.0175	0.2118	0.7377	/
L3.18	0.7854	0.8359	0.7982	0.134	0.0317	0.0987	0.6846	0.5992	0.7492	/
L3.26	0.8456	0.4629	0.9045	0.0836	0.0237	0.1182	0.6801	0.65	0.813	/
L3.71	0.6462	0.6524	0.8482	0.0629	0.0225	0.0601	0.7869	0.7334	0.7776	/
L3.72	0.1056	0.1517	0.3924	0.5038	0.8173	1.089	0.1108	0.312	0.8562	/
L3.83	0.0607	0.1447	0.3372	0.6527	0.8569	0.956	0.0201	0.287	0.7767	/
L3.87	0.8764	0.851	0.8745	0.9477	0.915	0.7637	0.4705	0.6	0.7894	0.087
L4.1	0.8905	0.9556	0.1811	0.3767	0.1314	0.5584	0.5734	0.528	0.8493	/
L4.111	0.0277	0.0684	0.7621	0.3366	0.2883	0.7972	0.1652	0.1244	0.8286	/
L4.113	0.7978	0.716	0.8649	0.0937	0.0323	0.0661	0.661	0.5787	0.7748	/
L4.124	0.7976	0.8225	0.1382	0.8427	0.776	0.8845	0.1706	0.3082	0.1059	/
L4.141	0.0127	0.0467	0.7517	0.2624	0.6332	0.696	0.4001	0.8395	0.7784	/
L4.194	0.8621	0.7125	0.9101	0.1178	0.0445	0.14	0.3217	0.7686	0.8792	/
L4.234	0.7394	0.786	0.8021	0.1572	0.0453	0.5141	0.6224	0.5491	0.7662	/
L4.264	0.0459	0.106	0.2849	0.9552	0.9004	0.8651	0.1785	0.2665	0.9197	/
L4.266	0.0178	0.0283	0.929	0.3155	0.8746	0.772	0.2326	0.6414	0.8846	/
L4.307	0.1069	0.1581	0.3298	1.0361	0.9454	0.9441	0.0939	0.2502	0.9891	/
L4.45	0.7868	0.7536	0.9081	0.1741	0.0691	0.0961	0.8254	0.6132	0.7988	/
L4.65	0.461	0.5848	0.7553	0.1056	0.0206	0.1786	0.3461	0.449	0.7855	/
L4.71	0.5349	0.6452	0.6601	0.0937	0.0412	0.1073	0.5257	0.701	0.7293	/
L4.77	0.1619	0.2342	0.2373	1.0234	0.9583	0.1378	0.162	0.368	0.979	/
L4.89	0.777	0.787	0.7678	0.1369	0.0228	0.0696	0.2025	0.499	0.6302	/
L4.93	0.868	0.9335	0.873	0.2669	0.1308	0.3264	0.3661	0.493	0.8467	/
L5.34	0.098	0.1118	0.3577	0.9098	0.8719	0.4213	0.1334	0.369	0.9274	/
L5.45	0.0911	0.1311	0.8852	0.408	0.4131	0.1792	0.089	0.227	0.9393	/
L5.6	0.1416	0.1626	0.8195	0.4121	0.5281	0.9021	0.1111	0.282	0.9388	/
L6.18	0.2506	0.3103	0.6378	0.4094	0.5732	0.2262	0.476	0.683	0.6095	/
L6.92	0.8612	0.7804	0.8646	0.9691	0.8648	0.786	0.5781	0.756	0.8388	0.1239
L6.170	0.8405	0.9274	0.8837	0.2054	0.0923	0.2977	0.608	0.5686	0.8028	/
S1.24	0.4592	0.4507	0.7169	0.1225	0.0285	0.1384	0.4396	0.5852	0.6848	/
S1.35	0.8727	0.9713	0.2323	0.9505	0.8503	0.7737	0.1426	0.36	0.0582	/
S1.42	0.0632	0.1257	0.3378	0.9262	0.8749	0.8611	0.2453	0.2644	0.8961	/
S2.114	0.8589	0.9908	0.2269	0.9271	0.9036	0.7656	0.1458	0.1097	0.0577	/
S2.116	0.6588	0.6945	0.8157	0.1661	0.0282	0.07	0.5476	0.5384	0.8652	/
S2.124	0.7915	0.7613	0.8142	0.1432	0.0294	0.1559	0.6394	0.5995	0.7664	/
S2.126	0.8579	0.9576	0.1273	0.9042	0.8607	0.8709	0.254	0.3328	0.0579	/
S2.129	0.8376	0.783	0.8522	0.1176	0.0332	0.2471	0.6874	0.5458	0.8257	/
S2.143	0.7249	0.4621	0.6892	0.1293	0.0894	0.1784	0.6875	0.5781	0.6825	/
S2.84	0.2403	0.3556	0.6263	0.5302	0.3606	0.7853	0.7851	0.5847	0.7394	0.681
S3.12	0.1126	0.1414	0.3983	0.6065	0.9139	0.9166	0.2001	0.2725	0.9569	/
S3.15	0.04	0.052	0.8891	0.2763	0.592	0.775	0.5115	0.6608	0.8511	/
S3.16	0.6606	0.7784	0.7971	0.0669	0.0131	0.0519	0.7095	0.5953	0.8144	/
S3.19	0.4752	0.8303	0.6804	0.624	0.5566	0.3555	0.3471	0.502	0.6306	0.1256
S3.34	0.592	0.8842	0.872	0.9097	0.8617	0.7796	0.6411	0.5674	0.7973	0.1898
S3.44	0.5993	0.6483	0.8961	0.0825	0.0198	0.1211	0.6742	0.5086	0.8116	/
S5.22	0.8741		0.9056	0.1032	0.0692	0.125	0.7743		0.9277	/
S5.31	0.7214	0.7773	0.8848	0.1204	0.0245	0.1165	0.672	0.5596	0.8661	/
S5.55	0.8589	0.0872	0.3523	0.9807	1.0025	1.0165	0.1464	0.374	0.0577	/

Supplementary Fig. S4: Epitope binning of bnAbs to 9 benchmark mAbs targeting 7 antigenic sites

a The epitope foodprints of S309 (left) and C022 (right). **b**The value shows in the table represented the comparation percentage that calculated by Data Analysis HT 9.0. Sensors immobilized prototype RBD bound bnAbs first and then detected the percentage epitope-known antibodies could bind. 30% were set as the positive comparation value and marked in red.

Supplementary Fig. S5

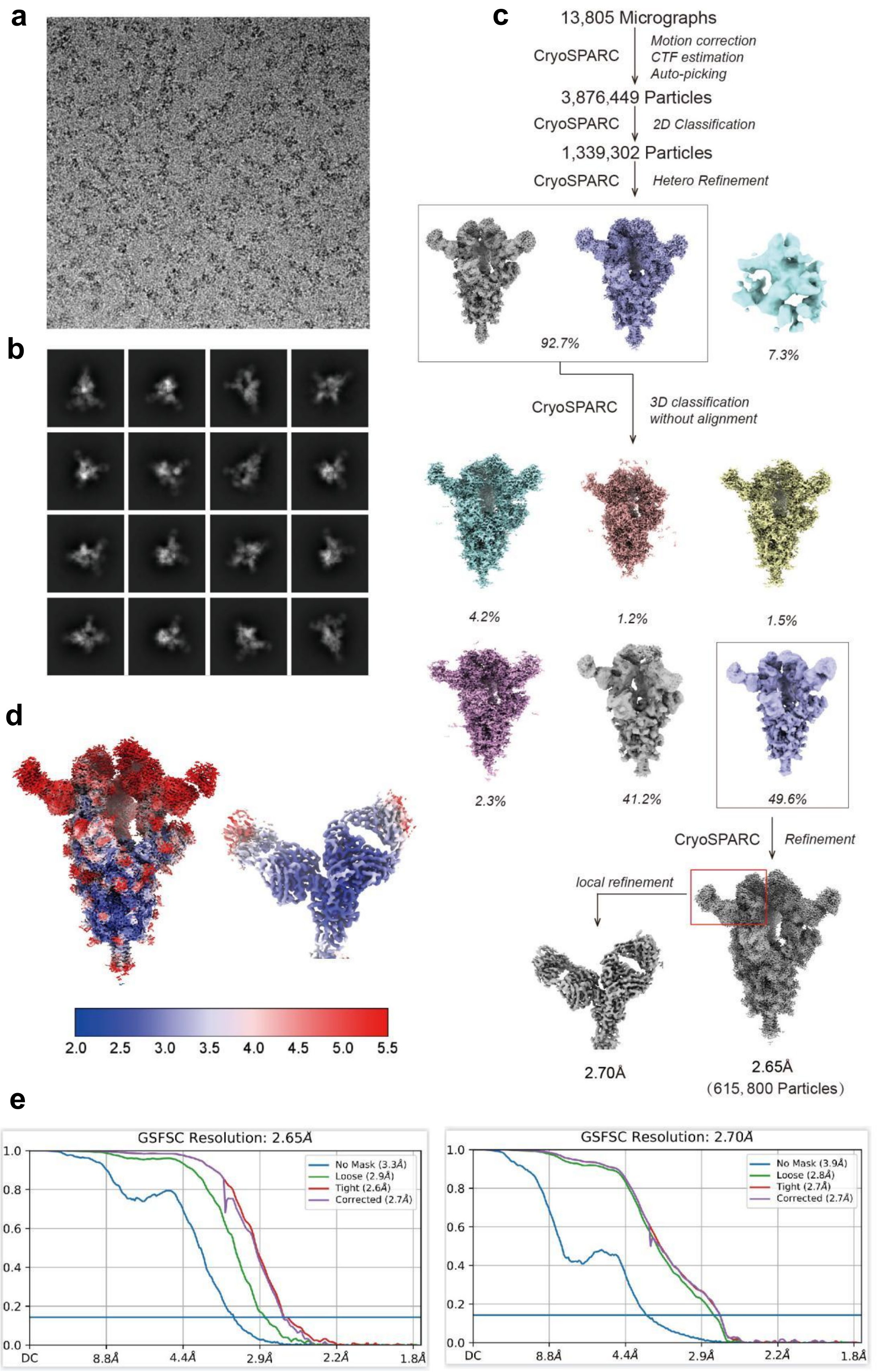

Supplementary Fig. S5: Cryo-EM data processing of Prototype S-L4.56-L5.34

a Representative cryo-EM micrograph of the Prototype S-L4.65-L5.34 sample. **b** 2D class average images of Prototype S-L4.65-L5.34 sample. **c** A brief workflow of cryo-EM image processing and reconstruction. **d** Cryo-EM map of Prototype S-L4.65-L5.34, and L4.65-RBD-L5.34 colored by local resolution (Å). **e** The FSC curves for the reconstructions.

Supplementary Fig. S6

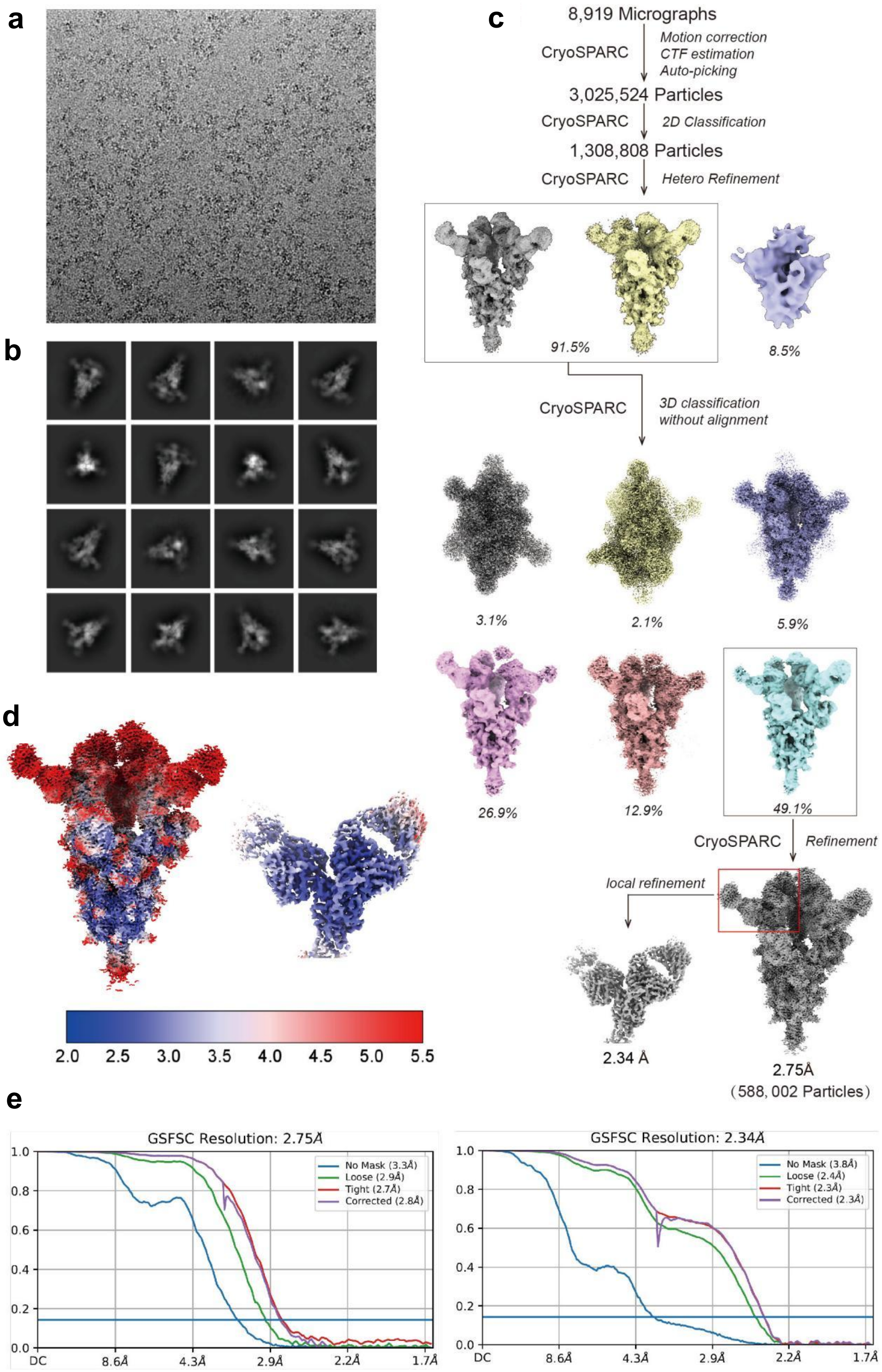

Supplementary Fig. S6: Cryo-EM data processing of BA.2 S-L4.56-L5.34

a Representative cryo-EM micrograph of the BA2 S-L4.65-L5.34. **b** 2D class average images of BA2 S-L4.65-L5.34. **c** A brief workflow of cryo-EM image processing and reconstruction. **d** Cryo-EM map of BA2 S-L4.65-L5.34, and L4.65-RBD-L5.34 colored by local resolution (Å). **e** The FSC curves for the reconstructions.

Supplementary Fig. S7

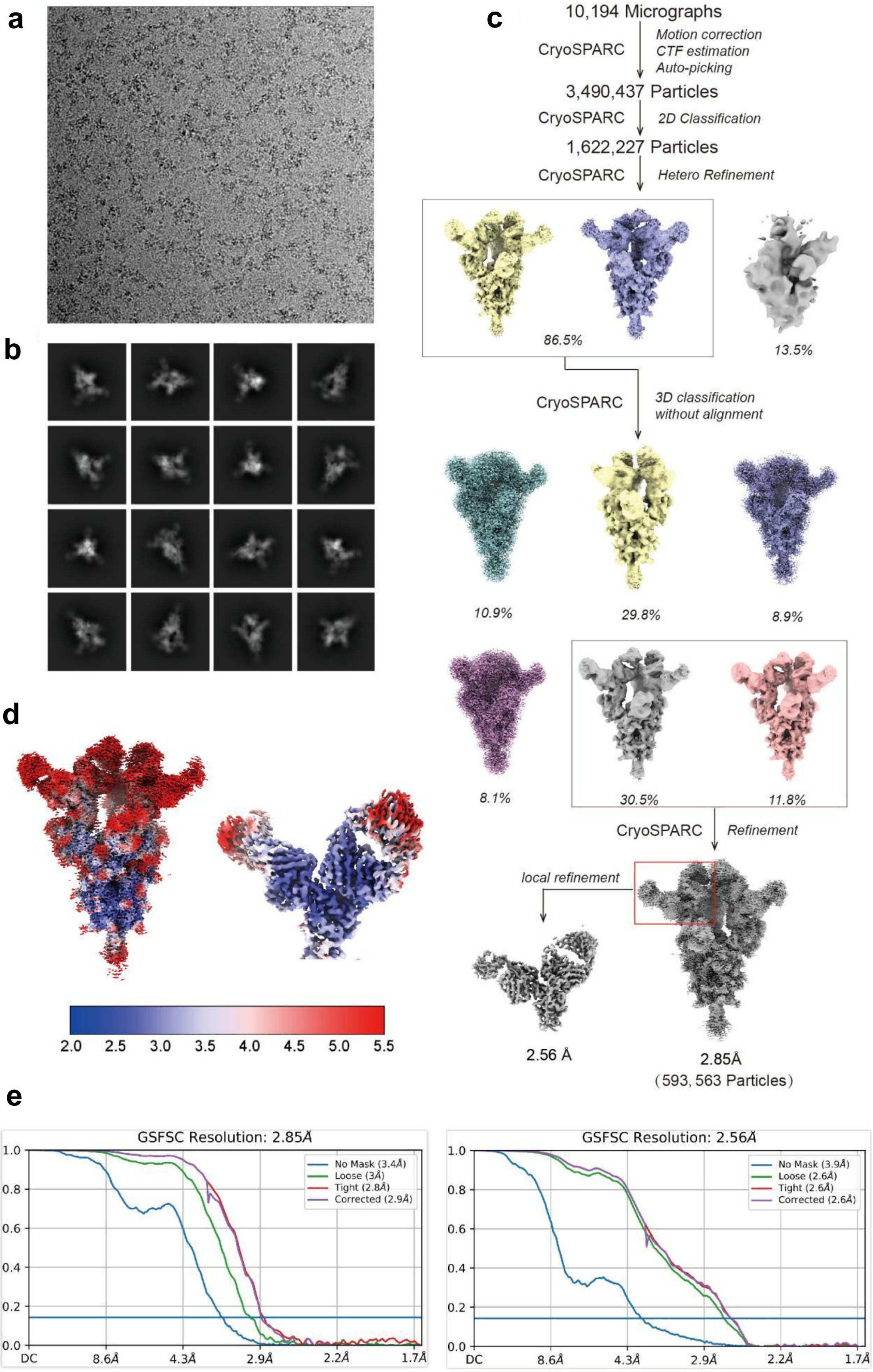

Supplementary Fig. S7: Cryo-EM data processing of BA.4/5 S-L4.56-L5.34

a Representative cryo-EM micrograph of the BA4 S-L4.65-L5.34. **b** 2D class average images of BA4 S-L4.65-L5.34. **c** A brief workflow of cryo-EM image processing and reconstruction. **d** Cryo-EM map of BA4 S-L4.65-L5.34, and L4.65-RBD-L5.34 colored by local resolution (Å). **e** The FSC curves for the reconstructions.

Supplementary Fig. S8

A

KD (M)	**PT**	**BA.2**	**BA.4**
L4.65	4.35E-11	<E-12	5.47E-12
L5.34	5.43E-12	1.67E-10	4.07E-10

B

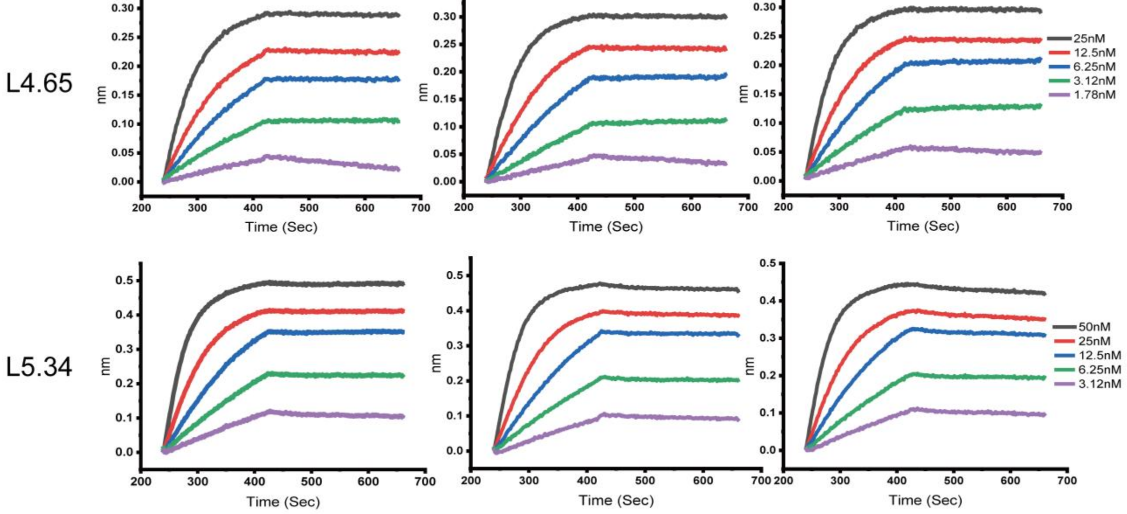

Supplementary Fig.8: Binding kinetic and affinity diagram of L4.65 and L5.34 against three RBD proteins

a Binding affinity of two antibodies bound to prototype RBD, BA.2 RBD and BA.4/5 RBD. **b** Prototype RBD, BA.2 RBD and BA.4/5 RBD bound to two antibodies. The antibody proteins were immobilized on the AHC sensors and then bound with gradient concentrations of RBD proteins by OctetRed 96. The binding profiles are shown with time (s) on the x axis and response value (nm) on the y axis.

Supplementary Table S1: Cryo-EM data processing and refinement statistics

	Prototype S -L4.65-L5.34	Prototype RBD -L4.65-L5.34
**Data collection and processing**		
Magnification	105k	105k
Voltage (kV)	300	300
Electron exposure ($e^{-}/Å^2$)	50	50
Defocus range (μm)	-1.0 to -2.0	-1.0 to -2.0
Pixel size (Å)	0.856	0.856
Symmetry imposed	C1	C1
Final particle images (no.)	615,800	615,800
Map resolution (Å)	2.65	2.70
FSC threshold	0.143	0.143
Map resolution range (Å)	2.0-5.5	2.0-5.5
**Refinement**		
Initial model used (PDB code)	6XCN	6XCN
Model resolution (Å)	2.65	2.70
Map sharpening *B* factor ($Å^2$)	-92.0	-66.8
Model composition		
Non-hydrogen atoms	44640	8292
Protein residues	5781	1082
Ligands	6	2
R.m.s. deviations		
Bond lengths (Å)	0.004	0.003
Bond angles (°)	0.685	0.733
**Validation**		
MolProbity score	1.66	1.65
Clashscore	7.30	5.88
Poor rotamers (%)	0.04	0.00
Ramachandran plot		
Favored (%)	96.14	95.24
Allowed (%)	3.86	4.76
Disallowed (%)	0	0

	BA2 S -L4.65-L5.34	BA2 RBD -L4.65-L5.34
**Data collection and processing**		
Magnification	105k	105k
Voltage (kV)	300	300
Electron exposure ($e^{-}/Å^2$)	50	50
Defocus range (μm)	-1.0 to -2.0	-1.0 to -2.0
Pixel size (Å)	0.84	0.84
Symmetry imposed	C1	C1
Final particle images (no.)	588,002	588,002
Map resolution (Å)	2.75	2.34
FSC threshold	0.143	0.143
Map resolution range (Å)	2.0-5.5	2.0-5.5
**Refinement**		
Initial model used (PDB code)	6XCN	6XCN
Model resolution (Å)	2.75	2.34
Map sharpening *B* factor ($Å^2$)	-118.7	-67.5
Model composition		
Non-hydrogen atoms	45516	8268
Protein residues	5837	1079
Ligands	6	1
R.m.s. deviations		
Bond lengths (Å)	0.008	0.003
Bond angles (°)	0.798	0.706
**Validation**		
MolProbity score	2.07	2.53
Clashscore	12.79	10.44
Poor rotamers (%)	0.00	7.09
Ramachandran plot		
Favored (%)	92.84	95.04
Allowed (%)	7.11	4.96
Disallowed (%)	0.05	0

	BA4 S -L4.65-L5.34	BA4 RBD -L4.65-L5.34
**Data collection and processing**		
Magnification	105k	105k
Voltage (kV)	300	300
Electron exposure (e⁻/Å^2)	50	50
Defocus range (μm)	-1.0 to -2.0	-1.0 to -2.0
Pixel size (Å)	0.84	0.84
Symmetry imposed	C1	C1
Final particle images (no.)	593,563	593,563
Map resolution (Å)	2.85	2.56
FSC threshold	0.143	0.143
Map resolution range (Å)	2.0-5.5	2.0-5.5
**Refinement**		
Initial model used (PDB code)	6XCN	6XCN
Model resolution (Å)	2.85	2.56
Map sharpening *B* factor (Å^2)	-121.7	-72.1
Model composition		
Non-hydrogen atoms	44355	8281
Protein residues	5745	1080
Ligands	3	1
R.m.s. deviations		
Bond lengths (Å)	0.013	0.003
Bond angles (°)	1.128	0.710
**Validation**		
MolProbity score	1.78	1.88
Clashscore	9.78	10.12
Poor rotamers (%)	0.64	0.11
Ramachandran plot		
Favored (%)	96.13	94.95
Allowed (%)	3.76	5.05
Disallowed (%)	0.11	0.00
